# Attitude of physiotherapists toward electronic health record in Croatia

**DOI:** 10.1186/s40945-019-0062-7

**Published:** 2019-10-22

**Authors:** Manuela Filipec, Gordana Brumini

**Affiliations:** 1 Department of Physical Medicine and Rehabilitation, Clinical Hospital, Sveti Duh 64, 10 000 Zagreb, Croatia; 2 Department of Medical Informatics, Rijeka University School of Medicine, Rijeka, Croatia

**Keywords:** Attitude, Physiotherapist, Electronic health record, Croatia

## Abstract

**Background:**

Electronic health record can facilitate everyday clinical practice of physiotherapists. The aim of this study is to determine attitude of physiotherapists towards implementation of information technology in their work and the differences in attitude in relation to gender, age, level of education and type of health institutions.

**Methods:**

This study is a cross-sectional survey of Croatian physiotherapists. The questionnaire ″Attitude of physiotherapists towards implementation of electronic health record included 12 items scored on a Likert-type scale from 1 to 5 and presented the award values as “Disagree”, “Neither agree nor disagree” and “Agree”. Croatian physiotherapists were (*n* = 267) recruited from 13 health care institutions. For analysis chi square test, t-test, one-way analysis of variance and as a post-hoc Tukey test were used.

**Results:**

Explanatory factorial analysis confirmed two factors: Satisfaction in the work of physiotherapists using computers (SAT) and Necessity of computers in the work of physiotherapists (NEC). Most physiotherapists agree on (SAT) (47.9%) and on (NEC) (51.3%). Male physiotherapists were significantly more likely to disagree with statements related to SAT (*p* < 0.001) and NEC (*p* = 0.035) than female physiotherapists. Physiotherapists aged between 46 and 55 years were significantly more like to disagree on NEC in comparison to all the other groups of participants (*p* < 0.001). Physiotherapists with secondary school degree were significantly more like to disagree on NEC as compared with participants with bachelor’s degree (*p* = 0.009), as well as in comparison with physiotherapists with a university degree (*p* = 0.002). Most of the physiotherapists who are employed in Clinical hospitals and in the Speciality hospital agree with that statement (all *p* > 0.05).

**Conclusion:**

The attitude of Croatian physiotherapists towards electronic health record differs according to the age, gender, level of education and type of health care institutions. This finding can facilitate implementation of electronic health record in physiotherapy.

**Trial registration:**

Not applicable.

## Introduction

Today’s information age and the rapid development of information and communication technologies have transformed the health care and the way of working in health care institutions. Physiotherapists nowadays work in the information technology (IT) environment and use computers and electronic health records in everyday clinical practice [1–3]. Documentation in physiotherapy is an essential part of the clinical process and the way of showing evidence of practice [3].

Electronically stored information in physiotherapy plays a role in the planning, assessment, intervention, evaluation, education and research [4]. Properly and timely retrieval of stored data has a significant impact on the quality of the process of physiotherapy in order to improve the quality of physiotherapy services [5]. Measuring of success for physiotherapy treatment, continuous monitoring of the patient, but also the evaluation of the treatment are imperative to keep the physiotherapy process documented and use this documentation as a tool to describe physiotherapy practice [6]. Documentation is a tool which describes physiotherapy assessment, outcomes, interventions and evaluation of physiotherapy process. The usage of electronic health records (EHR) in physiotherapy provides continuous insight into conditions of the patient, the availability of data, continuity of physiotherapy care, chronological overview of the results and evaluation as well as the database for training physiotherapists and research in physiotherapy [2].

Indeed, EHR preserves information about demographics, medications, vital signs, past medical history, immunisations, laboratory data, radiology reports and physiotherapy proces. It allows information on patient’s functional status and progress and could be used to monitor the effectiveness of physiotherapy interventions (2). Beside the clinical outcome (improvements in the quality of care) EHR contains the organisational issues (such as financial and operational performance) and social issues (including being more able to conduct research and eventually achieve improved population health) (2). EHR serves as a database repository that is available to physiotherapists and all team members.

International Association for Medical Informatics indicates: to successfully keep pace with the rate of growth of medical knowledge, it is necessary to use a new methodology, information management and modern information and communication technologies. Health professionals who can handle medical IT will systematically and promptly process the medical information, as well as competently and responsibly use the modern information and communication technology in their daily work [7].

Clinical decision-making in physiotherapy should be based on arguments, facts and information that must be available, accurate, current, timely, secure and unchangeable [8]. The purpose of the application of the electronic health record in physiotherapy is the improvement of service quality and consistency in the clinical approach to patients and therefore upgraded patients’ safety. EHR provides better communication between all participants in health care through a central data management of patients, both in the primary as well as secondary and tertiary services. It enables patients to fully access [8] their health care records in a fast and secure way and to obtain the information about the service and quality of the physiotherapy process. EHR also facilitates more efficient administration, documentation and spending of funds [2, 9].

Electronic health record provides an easily accessible, reliable storage, manageable usage of health information of the patient, reviews of the history of the patient’s health status, sharing of patient’s information among authorized participants in the treatment of the patient and the source of data for National register and statistics. Therefore, it improves the quality and efficiency of the process of physiotherapy [9].

In physiotherapy practice, an effective and efficient decision-making is essential. Shaping an appropriate clinical decision requires the right information at the right time in the right format, although clinicians are often faced with redundant information that is ambiguous, incomplete and poorly organized [10]. Humans are an imperfect data processor, and therefore unclear or large amount of information often complicates the decision-making process. When deciding clinicians are faced with an enormous amount of information, they can be particularly susceptible to errors or omissions. Computers are tireless and accurate data processors which supplement the knowledge of clinicians and using electronic data leads to improved outcomes of physiotherapy.

Despite the promotion of information technology, adoption and implementation of EHR in physiotherapy are limited. Advantages of EHR in physiotherapy are organisational, like a larger possibility of documentation, improvement of operational efficiency, better interdepartmental communication, improvement of data accuracy and easy access to data for further research [11, 12]. Disadvantages of application EHR in physiotherapy are modifications of modes and behavior, software or hardware, training of personnel and integration analysis of the working process in the system design [13, 14]. Attitude towards EHRs is important because it can facilitate or disrupt implementation of EHR and influence the desire for learning in staff, which is the key to successful implementation of EHR [2].

This study aims to determine what is the attitude of Croatian physiotherapists towards the implementation of electronic health records (EHR) and are there any differences in attitude towards implementation of electronic health records in physiotherapy in relation to gender, age, level of education and type of health institutions physiotherapists work at.

## The method

### The design

This cross-sectional study used survey to detect physiotherapist’s attitude towards electronic health record in Croatia. The survey was conducted in Croatia during the period February to May 2014.

### Participants

The participants consented to complete the questionnaire after reading an information sheet. The study recruited physiotherapists from two Clinical Hospital Centers (CHC): Clinical Hospital Center Osijek in Osijek, Clinical Hospital Center Rijeka in Rijeka; two Clinical Hospitals (CH): Clinical Hospital Dubrava in Zagreb, Clinical Hospital “Sveti Duh” in Zagreb; seven General Hospitals (GH): General Hospital “Dr. Josip Bencevic” in Slavonski Brod, General Hospital “Dr. Tomislav Bardek” in Koprivnica, General Hospital Zadar in Zadar, General Hospital Dubrovnik in Dubrovnik, General Hospital Pula in Pula, General Hospital Gospic in Gospic, General Hospital Cakovec in Cakovec and two Specialty Hospitals (SP): Clinic of Orthopaedic Surgery Lovran in Lovran and Thalassotherapia - Special Hospital for medical Rehabilitation of heart, lung, and rheumatism in Opatija.

### The questionnaire

The data were collected using the original questionnaire created for this research. The questionnaire consists of three parts (Additional file 1). The first part consists of two questions and demographic data about subjects, their age and gender. The second part consists of two questions and includes the data subjects about the level of education and qualifications and the employment status. The third part of the questionnaire consists of 12 items. Some of them were partly based on the experience gained in conducting similar researches [15–18], and some were partly supplemented with new items based on consultation with two experts in the field testing of attitudes of health professionals. Newly added items are 2, 4, 5, 7, 8, 9 and 12. The answers were offered on a Likert-type scale from 1 to 5, where 1 indicates “strongly disagree”, 2 “disagree”, 3 “neutral”, 4 “agree” and 5 “strongly agree”.

### Data analysis

Reliability and validity of the questionnaire have been tested. To test structural validity explanatory factor analysis (EFA) was used, utilizing principal component analysis and Guttman - Kaiser criterion for limiting factor extraction. The basic solution was transformed by oblimin rotation. The reliability of each factor was calculated using Cronbach’s alpha coefficient. Internal consistency reliability has been estimated. Internal consistency is measured with Cronbach’s alpha, a statistic calculated from the correlations (Pearson Correlation) between items. Statistical analysis of data was computed using IBM SPSS Statistics, version 21 (Armonk, NY: IBM Corp. 2012). Significance of differences among physiotherapists according to age and gender, education and gender and state of employment and gender are determined by the chi square test. The statistical significance of differences in physiotherapists’ attitude towards the implementation of electronic health records according to gender was determined by t-test. The differences in attitude towards the implementation of the electronic health record according to the age and education and type of health institutions were determined by one-way analysis of variance (ANOVA) and as a post-hoc Tukey test was used. All results were considered significant at *p* < 0.05.

## Results

In total, the survey included 267 Croatian physiotherapists (24.0% of them worked at Clinical Hospital Centers, 21.3% at Clinical Hospitals, 41.2% at General Hospitals and 13.5% at Specialty Hospitals. The response rate of physiotherapists who participated in the study was 78.3% (Table 1).

**Table 1:** Response rate physiotherapists who participated in the study

Type of health institution	N of all fully completed questionnaires / N of all physiotherapists in these institutions	Response rate^a^ %
Clinical hospital center (CHC)	64//90	71.1
Clinical hospital (CH)	57/69	82.6
General hospital GH)	110/135	81.4
Speciality hospital (SH)	36/41	87.8
All	267/341	78.2

^a^response rate is calculated N of all fully completed questionnaires / N of all physiotherapists in these institutions

### Data about responders

The average age of physiotherapists was 40.5 ± 11.8 years (range 20–64). The results indicated a significant difference in age (*p* < 0.001) in relation to the type of health institutions they work at. Also, the results indicated a significant difference of distribution of male and female physiotherapists (*p* = 0.009) in Clinical hospitals (Table 2).

**Table 2:** Distribution of physiotherapists in relation to age, gender and level of education according to type of health institutions (data are expressed as number (%)

	Type of health care institutions					Statistic p
	CHC (*n* = 64)	CH (*n* = 57)	GH (*n* = 110)	SH (*n* = 36)	ALL (*n* = 267)	
Age /years /*x* ± SD	37. 7 ± 12.0	40.7 ± 10.3	42.1 ± 12.0	40.1 ± 12.8	40.5 ± 11.8	<0.001*
Gender/N(%)						
Female	42 (65.6)	44 (77.2)	81 (73.6)	27 (75.0)	194 (72.6)	0.170
Male	22 (34.4)	13 (22.8)	29 (26.4)	9 (25.0)	73 (27.4)	0.294
Statistic p	0.465	0.009	0.423	0.964	0.391	
Level of education/N(%)						
Secondary school	31 (48.4)	4 (7.0)	37 (33.6)	8 (22.2)	80 (29.9)	0.178
Bachelor’s degree	29 (45.3)	43 (75.4)	66 (60.0)	23 (63.9)	161 (60.3)	0.257
University degree	4 (6.3)	10 (17.6)	7 (6.4)	5 (13.9)	26 (9.8)	0.125
Statistic p	0.080	0.019	0.389	0.109	<0.005	

^*^Post-hoc test showed differences in age between physiotherapists CHC and the SH (*p* = 0.025)

The results indicated a significant difference in the distribution of all physiotherapists in relation to the degree of education (*p* < 0.005). When considering physiotherapists in each type of health care institution, there is statistical significance of distribution of physiotherapists according to the level of education in the CH (*p =* 0.019). The results does not indicated a significant difference in the distribution of physiotherapists considering other types of health institutions and level of education (all *p* > 0.05) (Table 2.).

#### EFA of the questionnaire

Two factors were obtained. The Factor I is called “Satisfaction in the work of physiotherapists using the computer” (SAT) and the Factor II is called “Necessity of computers in the work of physiotherapists” (NEC). The results are interpreted according to the Likert scale as “agree”, “neither agree nor disagree” and “disagree”.

The Factor I consists of eight statements (from 5 to 12). If SAT presented the award values of 8–18, it was considered as “disagree”, between 19 and 29 as “neither agree nor disagree”, and 30 to 40 as “agree”. The Factor II included three statements (from 1 to 3). If NEC presented the award values from 3 to 6 it was considered as “disagree”, 7 to 10 - “neither agree nor disagree”, and 11 to 15 - “agree”.

The statement 4, not fitting any of the two factors, it was excluded from the subsequent analysis.

SAT interprets 46.9% of the total variance and NEC interprets 9.8%. For SAT α is 0.900; and for NEC α = 0.676.

### Attitude of physiotherapists

It has been shown that the majority of physiotherapists in all types of health care institutions were more likely to „neither agree nor disagree “(*p*<0.001) with SAT. The majority participants were more like to “agree” with NEC in the CH, GH and SH (all *p* < 0.001). However, physiotherapists who were more like to “neither agree nor disagree” are significantly prevalent than physiotherapist who “disagree” and “agree” in the CHC (*p* < 0.001) (Table 3.).

**Table 3:** Satisfaction in the work of physiotherapists using the computers (SAT) and Necessity of computers in the work of physiotherapists (NEC) according to the type of health institution

	Type of health care institution					Statistic p
	CHC	CH	GH	SH	ALL	
SAT N (%)						
Disagree	14 (21.8)	16 (28.1)	27 (24.5)	5 (13.9)	62 (23.2)	0.017
Neither agree nor disagree	33 (51.6)	30 (52.6)	55 (50.0)	19 (52.8)	137 (51.3)	0.007
Agree	17 (26.6)	11 (19.3)	28 (25.5)	12 (33.3)	68 (25.5)	0.256
Statistic p	<0.001	<0.001	<0.001	<0.001	<0.001	
NEC N (%)						
Disagree	9 (14.1)	4 (7.0)	16 (14.5)	0 (0)	29 (10.9)	0.014
Neither agree nor disagree	33 (51.6)	26 (45.6)	42 (38.2)	5 (13.9)	106 (39.7)	0.011
Agree	22 (34.3)	27 (47.4)	52 (47.3)	31 (86.1)	132 (49.4)	0.047
Statistic p	<0.001	<0.001	<0.001	<0.001	<0.001	

The SAT differed only in relation to gender, male physiotherapists being significantly more likely to agree than female physiotherapists (*p* < 0.001). Coversely, the NEC differed according to age, gender and health care institutions. There were higher proportions of responders who disagreed of NEC statements among male physiotherapists than among female physiotherapists (*p* < 0.001); among physiotherapists aged between 46 and 55 years in comparison to all the other groups of participants (*p* < 0.001). Differences were also found according to level of education (*p* = 0.035), physiotherapists with secondary school being more likely to disagree than physiotherapists with bachelor’s degree (*p* = 0.009) or with a university degree (*p* = 0.002) (Table 4.).

**Table 4:** Satisfaction in the work of physiotherapists using the computers and necessity of computers in the work of physiotherapists in relation to gender, age, level of education and type of health care institution

	Satisfaction in the work of physiotherapists using the computers				Necessity of computers in the work of physiotherapists			
	N	*x* ± SD	F	p	N	*x* ± SD	F	p
Gender								
Female	194	23.0 ± 7.0		<0.001	194	10.0 ± 2.0		0.035
Male	73	27.0 ± 6.0			73	11.0 ± 2.0		
Age /years								
20–25	35	26.0 ± 7.0	1.6	0.160	35	11.0 ± 2.0	6.0	<0.001^*^
26–35	73	25.0 ± 6.0			73	11.0 ± 2.0		
36–45	58	24.0 ± 7.0			58	10.0 ± 2.0		
46–55	73	22.0 ± 7.0			73	9.0 ± 3.0		
56–65	28	25.0 ± 8.0			28	10.0 ± 3.0		
Level of education								
Secon.school	80	23.0 ± 7.0	1.1	0.327	80	9.0 ± 2.0	7.5	<0.001^**^
Bachel. deg.	161	24.0 ± 6.0			161	10.0 ± 2.0		
Univer. deg	26	25.0 ± 8.0			26	11.0 ± 2.0		
Type of health care institution								
CHC	64	24.9 ± 8.0	1.5	0.227	64	10.1 ± 3.0	5.7	<0.001^***^
CH	57	23.3 ± 7.0			57	11.1 ± 2.0		
GH	110	24.5 ± 7.4			110	10.0 ± 2.9		
SH	36	26.6 ± 6.0			36	12.0 ± 1.6		

^*^Post-hoc test showed that physiotherapists aged 46–55 had different attitude towards the necessity of computers than physiotherapists aged 20–25 (*p* < 0.005), 26–35 (*p* < 0.001) or 36–45 (*p* = 0.003)

^**^The post hoc test showed that physiotherapists with secondary school had different opinion about the necessity of computers compared to physiotherapists with bachelor’s degree (*p* = 0.009) or university degree (*p* = 0.002)

^***^The post hoc test showed differences in attitude towards the necessity of computers between physiotherapists working in the GH and physiotherapists working in the CH (*p* = 0.021) or in the SH (*p* = 0.003) and between physiotherapists working in the CHC and physiotherapists working in the SH (*p* = 0.037)

## Discussion

Our study showed that most Croatian physiotherapists “neither agree or disagree” with SAT and NEC. This result point out the indifference of Croatian physiotherapists to accept information technology in daily clinical work. and desire to improve the physiotherapy care. Our results are not consistent with other studies on the attitude of health professionals towards the implementation of EHR [18, 19] and indicate that most of Croatian physiotherapists are not satisfied with implementation of EHR in everyday work [20, 21]. The reason for this is perhaps that most physiotherapists in Croatia do not use computers (because they do not have them) in everyday work, and the use of new technologies causes discomfort, dissatisfaction and reduced readiness to accept EHR.

Statistical significance of differences in attitude of Croatian physiotherapists towards the implementation of EHR in relation to type of health institutions for SAT indicates practical implication in physiotherapy. Practical implication derived from this study showed that physiotherapists should be ready to change personal attitude towards computers and modes of documentation in daily work and improve computer skills if want to use EHR. They also should be willing to implement information technology in clinical practice. Using computers will contribute to greater satisfaction in the work of physiotherapists. Physiotherapists should not be discouraged by the fear of confronting new tools, personal IT ignorance, difficulties, mistakes and errors when working at a computer. The present study implies that Croatian physiotherapists should adopt strategies to change their attitude to positive towards implementation of EHR. This change can be induced through workshops about the possibilities and advantages of the practical application of EHR in physiotherapy and exchange of experiences of physiotherapists working at various types of health care institutions.

Statistical significance of differences in attitude of Croatian physiotherapists towards the implementation of EHR in relation to type of health institutions for NEC indicates a willingness to improve physiotherapy clinical practice. Traditionally, physiotherapists use a paper health record and unstructured data entry format that has little visibility and are often illegible because of handwriting, more time-consuming and easy to lose. Advantages of using an EHR in clinical practice of physiotherapists are reflected in improving and facilitating communication with team members, the availability of medical data in one place, easier documentation, transparency, accessibility, data recovery and database for future research in physiotherapy [22, 23]. These advantages provide physiotherapists a complete picture of the patient’s status which enables the physiotherapist to devote more time to analyzing the results of the assessment and planning appropriate interventions.

However, adequate information technology and its availability, as well as accompanying support of IT services and financial resources are often the causes of difficulties for the successful implementation of EHR [24].

The attitude of Croatian physiotherapists towards implementation of electronic health records and NEC varies significantly according to age, gender and degree of education. Contrary to older physiotherapists, physiotherapists aged 46–55 are significantly less like to “agree” on NEC than their younger colleagues. This is consistent with the results of other studies [25–27]. One study indicates that age is not associated with the attitude of physicians towards EHR, but the experience of working on a computer and the satisfaction of working on a computer are [20].

Although our study identifies differences in attitude of Croatian physiotherapists towards using EHR in relation to gender, it is reported in other studies that gender is not associated with attitude of health professionals towards implementation of EHR [19, 20, 25]. The reasons for this may be numerous: perhaps a too small sample, the difference in age or degree of education.

In present study, Croatian physiotherapists with a lower degree of education are more likely to agree less on the implementation of EHR than physiotherapists with a high level of education. These results and the results of other studies indicate that age and level of education have an effect on taking the attitude of physiotherapists, nurses and physicians towards the implementation of EHR [21, 27, 28]. The reason for this is probably a lower level of education in elderly health professionals, less usage of computers in their daily work as well as the resistance and unwillingness to adopt new skills later in life, and perhaps discomfort due to ignorance of computer usage.

The limitations of this study are sample size and the number of included institutions, as well as a lack of comparison of attitudes towards EHR between Croatian physiotherapists and physiotherapists in other countries. Also, the lack of analysis to show which variables (age, gender, level of education, etc.) are independently associated with attitude towards EHR, the lack of data on senior physiotherapists, specialization and availability of information technology. Probably the work place or the type of department and the challenge of the work of Croatian physiotherapists in different health care institutions affect attitude towards implementation of EHR. The Clinical hospital centers, due to the higher education teaching and scientific research conducted there, require the use of computers, which certainly contributes to a more positive attitude towards the implementation of EHR compared to the General Hospitals. The Clinical hospital centers have multiple specialities and fields of work in comparison to Clinics which are aimed at specialist and consultation activities in one area. Quite possibly the diversity of specialities in one medical institution contributes to a more positive attitude compared to the attitude of physiotherapists from one speciality in a health care institution.

## Conclusion

The present study indicates differences in attitude of Croatian physiotherapists towards EHR in relation to gender, age, level of education and type of health care institution. This suggests the importance and necessity of education of physiotherapists to improve computer skills and raise the motivation for using computers in daily clinical work. This study is relevant to facilitate the implementation of EHR in physiotherapy, but more research on larger sample is necessary.

## Additional file

**Additional file 1.** The questionnaire "Atttitude of physiotherapists toward implementation of electronic health record.

## Data Availability

The datasets used and/or analyzed during the current study available from the corresponding author on reasonable request.

## Abbreviations

ANOVA: One-way analysis of variance
CH: Clinical Hospitals
CHC: Clinical Hospital Centers
EFA: Explanatory factor analysis
EHR: Electronic health record
GH: General Hospitals
IT: Information technology
SP: Speciality Hospitals
