# Supplementary material for: Attitude of physiotherapists toward electronic health record in Croatia
Source: Arch Physiother. 2019 Oct 22;9:10. doi: 10.1186/s40945-019-0062-7 (PMC6805644; doi:10.1186/s40945-019-0062-7)
Supplement: Supplementary file 1 — Additional file 1. The questionnaire "Atttitude of physiotherapists toward implementation of electronic health record. [file 40945_2019_62_MOESM1_ESM.docx]

Additional file 1

The questionnaire presented to Croatian physiotherapists was in Croatian.

Appendix 1 has been translated into English from Croatian language.

With the questions begin "Entering data into a computer", we would like to say that EHR facilitates work of physiotherapists. Precisely, we mean that functions and uses of EHRs could increase the quality of physiotherapy services.

The questions about "working on a computer" or "computer work" are in comparison to the paper record. We mean that it is easier to entered patient data by computer rather than keep paper record.

These explanations were provided to the responders with this instruction: The questions begin "Entering data into a computer","working on a computer" or "computer work" relate to whether the application of the EHR record in comparison to a paper record is considered to take less time for the documentation and allows more time to take an anamnesis, assess of the functional status and conduct the exercises, thus facilitating everyday work and improving the quality of the physiotherapy process. The question “Working on a computer reduces the time spent with the patient” relates to the reduction of the patient's time at each arrival at the physiotherapist because the EHR shows all the current physiotherapy data, which facilitates the physiotherapist's work.

The questionnaire “Attitude of physiotherapists toward implementation of electronic health record”

1. Age: years
2. Gender: M F
3. Degree of education:
   1. secondary school
   2. bachelor's degree
   3. university degree
4. Are you employed in the profession?
   1. Yes
   2. No

Please evaluate the extent to which you agree with each statement and mark your answer. The numbers indicate the following:

1. strongly disagree
2. disagree
3. neither agree nor disagree
4. agree
5. strongly agree

| 1. | A computer is essential in the daily work of physiotherapists. | 1 | 2 | 3 | 4 | 5 |
| --- | --- | --- | --- | --- | --- | --- |
| 2. | Physiotherapists are sufficiently educated (having the necessary information technology skills) to work on a computer. | 1 | 2 | 3 | 4 | 5 |
| 3. | A computer facilitates entry of patient’s data. | 1 | 2 | 3 | 4 | 5 |
| 4. | Working on a computer reduces the time spent with the patient. | 1 | 2 | 3 | 4 | 5 |
| 5. | Entering data into a computer allows a physiotherapist to pay more attention to the patient during physiotherapy assessment and intervention issues. | 1 | 2 | 3 | 4 | 5 |
| 6. | Computers are reliable. | 1 | 2 | 3 | 4 | 5 |
| 7. | The daily usage of computers improves work of physiotherapists. | 1 | 2 | 3 | 4 | 5 |
| 8. | Entering data into a computer provides an easier and better communication with the patient. | 1 | 2 | 3 | 4 | 5 |
| 9. | Entering data into a computer enables an easier and faster communication of the physiotherapist with other team members. | 1 | 2 | 3 | 4 | 5 |
| 10. | Work productivity increases by using a computer. | 1 | 2 | 3 | 4 | 5 |
| 11. | Computer work is inspiring and provides satisfaction in the work of physiotherapists. | 1 | 2 | 3 | 4 | 5 |
| 12. | Entering data into a computer increases the quality of physiotherapy services. | 1 | 2 | 3 | 4 | 5 |
